# Conformational Shifts of Stacked Heteroaromatics: Vacuum vs. Water Studied by Machine Learning

**DOI:** 10.3389/fchem.2021.641610

**Published:** 2021-03-26

**Authors:** Johannes R. Loeffler, Monica L. Fernández-Quintero, Franz Waibl, Patrick K. Quoika, Florian Hofer, Michael Schauperl, Klaus R. Liedl

**Affiliations:** Center of Molecular Biosciences Innsbruck, Institute of General, Inorganic and Theoretical Chemistry, University of Innsbruck, Innsbruck, Austria

**Keywords:** machine learning, stacking, solvation, heteroaromatics, ANI

## Abstract

Stacking interactions play a crucial role in drug design, as we can find aromatic cores or scaffolds in almost any available small molecule drug. To predict optimal binding geometries and enhance stacking interactions, usually high-level quantum mechanical calculations are performed. These calculations have two major drawbacks: they are very time consuming, and solvation can only be considered using implicit solvation. Therefore, most calculations are performed in vacuum. However, recent studies have revealed a direct correlation between the desolvation penalty, vacuum stacking interactions and binding affinity, making predictions even more difficult. To overcome the drawbacks of quantum mechanical calculations, in this study we use neural networks to perform fast geometry optimizations and molecular dynamics simulations of heteroaromatics stacked with toluene in vacuum and in explicit solvation. We show that the resulting energies in vacuum are in good agreement with high-level quantum mechanical calculations. Furthermore, we show that using explicit solvation substantially influences the favored orientations of heteroaromatic rings thereby emphasizing the necessity to include solvation properties starting from the earliest phases of drug design.

## Introduction

Binding between targets and small molecule drugs depends on a small set of specific interactions (Bissantz et al., 2010). In structure-based drug design, the main goal is to optimize a small molecule to make use of all possible interaction sites provided by the protein's binding pocket (Bissantz et al., 2010; Kuhn et al., 2011). Computer simulations of protein ligand complexes and various approaches to predict the binding free energy are readily used in the drug design process (Chang et al., 2007; Chodera et al., 2011; Mobley and Klimovich, 2012; Limongelli et al., 2013; Hansen and Van, 2014). However, certain interactions, e.g., π-π stacking of heteroaromatics, are not properly parametrized in modern force fields to reliably make free energy estimations. Yet, these interactions play a major role in drug design (Burley and Petsko, 1985; Meyer et al., 2003; Williams et al., 2003; Adhikary et al., 2019). Heteroaromatic moieties or cores are found in the majority of drug molecules (Meyer et al., 2003; Wang et al., 2017) as they present ideal modification sites and allow for unique interactions, i.e., stacking (Meyer et al., 2003; Salonen et al., 2011). Stacking can occur as π-π (Huber et al., 2014), halogen-π (Wallnoefer et al., 2010), amide-π (Harder et al., 2013; Bootsma and Wheeler, 2018), cation-π (Gallivan and Dougherty, 1999), and even anion-π (Wheeler and Bloom, 2014) interactions.

The state-of-the-art approach to estimate stacking interactions and to identify favorable geometries is the application of high-level quantum mechanical calculations. This can either be done by using a grid-based approach (Huber et al., 2014; Bootsma et al., 2019) or by using descriptors derived from high-level quantum mechanical calculations (Bootsma and Wheeler, 2011). However, to obtain interaction energies via a grid-based approach, numerous calculations have to be performed and molecules are restrained in single-point calculations (Huber et al., 2014). Furthermore, these calculations are almost exclusively performed in vacuum or implicit solvent. Nevertheless, several studies have investigated the effect of solvation on stacking interactions and the resulting implications on thermodynamic properties (Kolár et al., 2011; Lee et al., 2019; Loeffler et al., 2020). In general, assessment of the desolvation penalty is crucial in drug design as it can reveal why certain molecules do not reflect the expected gain in binding affinity (Biela et al., 2012; Dobiaš et al., 2019; Loeffler et al., 2020). Therefore, a combination of approaches is inevitable to understand the energetics of molecules and to interpret and optimize SAR studies (Loeffler et al., 2020). Since quantum mechanical calculations come with an extreme computational cost, several ways to minimize calculation time have been developed, including fragmentation (Kitaura et al., 1999), semi-empirical methods (Dewar et al., 1985; Elstner, 2006; Stewart, 2009) and recently machine learning approaches (Smith et al., 2017, 2018). Machine learning is a powerful tool and has already been applied to address various challenges in chemistry, e.g., the prediction of binding affinity (Nguyen et al., 2019), atomic forces, nuclear magnetic resonance shifts (Ghosh and Hammes-Schiffer, 2015), and even the prediction of reaction pathways (Jiang et al., 2016). Additionally, it has been shown, that machine learning approaches allow substantially faster predictions of quantum mechanically calculated potential energy surfaces (Chmiela et al., 2017; Schütt et al., 2017; Smith et al., 2018; Yao et al., 2018), geometries and atomic charge models (Smith et al., 2017). In recent years, potentials based on deep neural networks have been developed and have already widely been applied to tackle several challenges, as they promise quantum accuracy at classical cost (Smith et al., 2017; Wang et al., 2018; Wang and Riniker, 2019; Xu et al., 2019; Ghanbarpour et al., 2020). These neural networks, in particular, the ANAKIN-ME (Accurate NeurAl networK engINe for Molecular Energies)—short ANI (Chmiela et al., 2017; Smith et al., 2017), have been trained to learn the potential energy surfaces. Similar to classical force fields, electrons are not treated explicitly in ANI. Additionally, the potential energy is calculated as a function of the geometric positions of atoms. In contrast, ANI does not use predefined properties such as atomic bonds, as in quantum mechanical calculations, and the energies in ANI are an artificial neural network. As the energy is not obtained by solving the Schroedinger equation, the computational effort of ANI is substantially reduced when compared to high-level QM calculations (Gao et al., 2020). From the potential energy surfaces of organic molecules in a transferable way, including both the conformational and configurational space, ANI is able to predict the potential energy for molecules outside the training set.

To investigate protein-ligand interactions molecular dynamics simulations are a standard tool in computational drug design (Michel and Essex, 2010). Usually additive force fields are used to study the dynamic properties of proteins (Tian et al., 2020). These approaches are well-suited to describe protein properties and give valuable insights to all kinds of properties including flexibility (Fernández-Quintero et al., 2019a) and plasticity of binding sites (Fernández-Quintero et al., 2019b) and protein-protein interfaces (Fernández-Quintero et al., 2020). Using computer simulations requires a balance between cost and accuracy. Compared to classical force fields, quantum-mechanical methods are highly accurate but computationally expensive and not feasible for large systems. In classical force fields, stacking interactions of heterocycles with aromatic amino acid sidechains are still challenging to describe (Sherrill et al., 2009; Prampolini et al., 2015). Therefore, studies on stacking interactions almost exclusively rely on high-level quantum mechanical calculations (Bootsma and Wheeler, 2011, 2018; Huber et al., 2014; Bootsma et al., 2019). The use of Machine learning combines the best of both approaches.

In this study we make use of the ANI potentials to calculate stacking interactions of heteroaromatics frequently occurring in drug design projects. We compare the calculated minimal energies with high-level quantum mechanical calculations in vacuum and in implicit solvation. Furthermore, we perform molecular dynamics simulations to generate an ensemble of energetically favorable and unfavorable conformations of heteroaromatics interacting with a truncated phenylalanine side chain, i.e., toluene, in vacuum and explicit solvation.

## Methods

### Data Set

**Figure 1:**
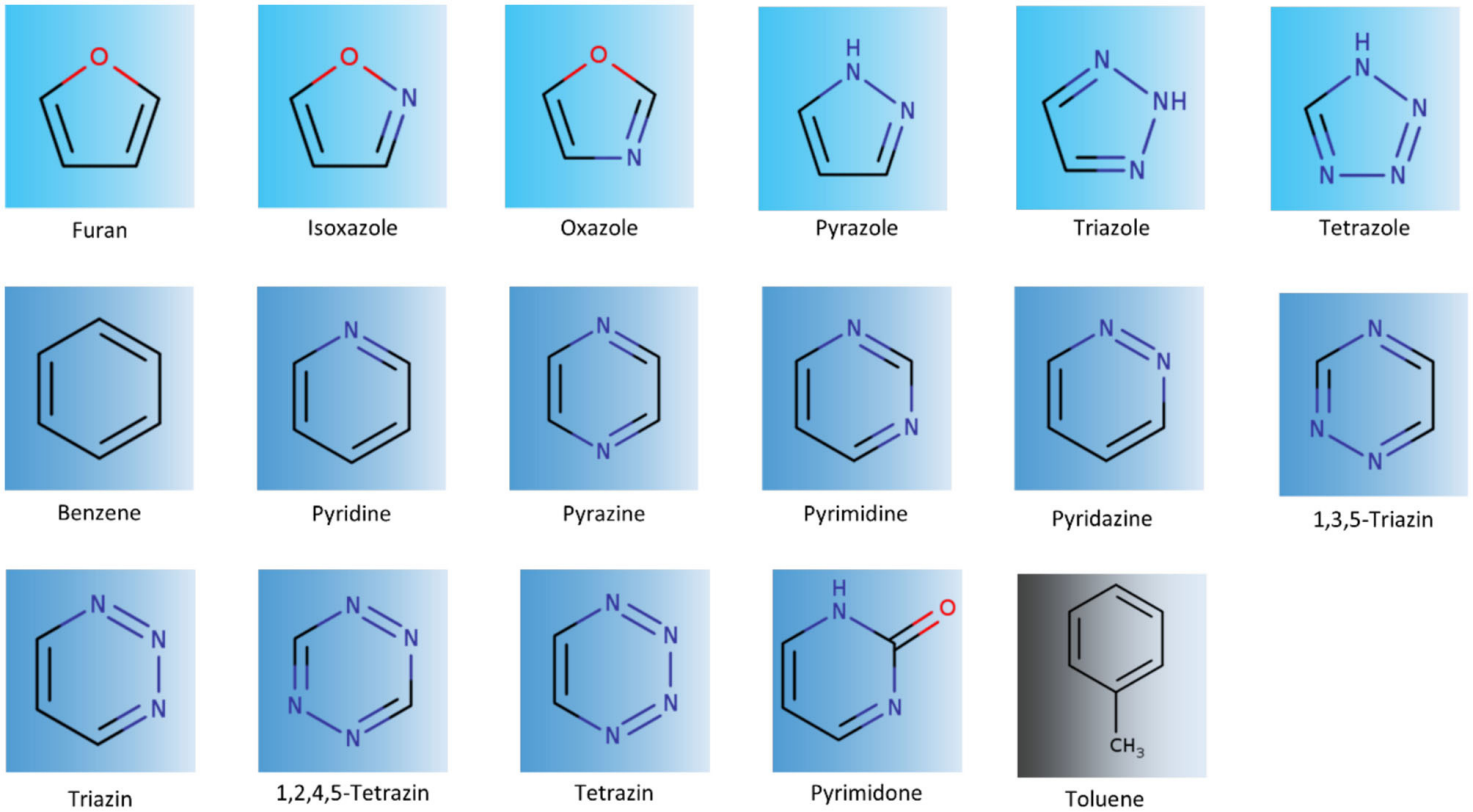
Overview of the analyzed aromatic molecules. Simulations were performed to investigate stacking interactions with toluene. We analyzed 5-membered heteroaromatics, furan, isoxazole, oxazole, pyrazole, triazole, and tetrazole. Furthermore, we simulated 6-membered rings, benzene, pyridine, pyrazine, pyrimidine, pyridazine, variants of triazine, and tetrazine, and pyrimidone.

The set of molecules investigated in this study frequently occurs in drug molecules (Salonen et al., 2011) and has already been investigated in previous publications to characterize their stacking properties using quantum mechanical calculations and molecular mechanics based calculations to estimate their respective solvation properties as monomers as well as complexes (Huber et al., 2014; Bootsma et al., 2019; Loeffler et al., 2019) (Figure 1).

### Quantum Mechanical Calculations

We followed the protocol recently introduced to perform energy optimization of heteroaromatics with toluene using Gaussian09 (Frisch et al., 2009) at the ωB97XD (Chai and Head-Gordon, 2008)/cc-pVTZ (Dunning, 1989) level. This combination has been benchmarked by Huber et al. (2014) and has been used in recent publications addressing similar questions (Loeffler et al., 2019, 2020). To better compare the geometries resulting from the simulations in water, we performed the geometry optimizations using an implicit water model. We used the polarizable continuum model, a reaction field calculation using the integral equation formalism (Tomasi et al., 2005) implemented in Gaussian09 (Frisch et al., 2009).

### ANI

This approach makes use of the Behler Parrinello symmetry functions to compute an atomic environment vector (AEV), $G_{i}^{X}$, which is composed of all elements, *G*_*M*_ probing regions of an atoms chemical surroundings. Each $E_{i}^{x}$ is then used as input to a single neural network potential. The energy of a molecule is calulated as the sum of all individual neural network potentials (Supplementary Figure 1).

The summation formalism to calculate *E*_τ_ shows two major advantages. Firstly, it allows fortransferability, and secondly, an even greater advantage is that due to the simple formalisma near linear scaling in computational complexity with added cores and/or GPUs is possible (Supplementary Figure 1).

### Simulation Setup

As starting structures for the simulations we used the minimum energy conformations provided in xyz-format in the Supplementary Material in the paper published by Bootsma et al. (2019). We solvated these conformations in a water box with a minimum wall distance of 10 Å using tleap resulting in approximately 1500 explicit water molecules (Case et al., 2018). To equilibrate the water box we performed a restrained equilibration allowing only the water molecules within the box to move as suggested in previous publications. During the equilibration performed with the AMBER simulation package we restrained the aromatic molecules to keep the geometry obtained from high-level QM calculations. The final frame of the equilibration was then used as starting structure of the production run. For each step of the simulations we calculated the forces and energies using ANI (Smith et al., 2017). To perform the simulations we used the atomic simulation environment (ASE) engine, protocol included in the Supplementary Material (Larsen et al., 2017). We used a timestep of 0.25 fs. To keep the temperature constant at 300 K we used the Langevin algorithm with a friction coefficient of 0.02 atomic units. We employed periodic boundary conditions in x, y, and z directions. We performed a short LBFGS (Head and Zerner, 1985) optimization before initiating the production runs of 100 ps. We performed this setup 10 times with different starting velocities for each heteroaromatic molecule.

### Vacuum Interaction Energies

To calculate the interaction energies in vacuum we performed the geometry optimization of the complexes and the respective monomers individually. These calculations were performed for force fields using MOE, for QM using Gaussian09 and for the ANI potentials using the ASE environment. The vacuum stacking interaction energies were then calculated according to the supermolecular approach as previously published. It has been shown that Counterpoise-corrections can result in distortions of the hypersurface (Liedl, 1998). Thus, and to allow for better comparability with the previous results no BSSE-correction was performed.

(1) $$\begin{array}{r} E_{interaction}=E_{complex}-E_{monomer\;A}-E_{monomer\;B} \end{array}$$

### Trajectory Analysis

**Figure 2:**
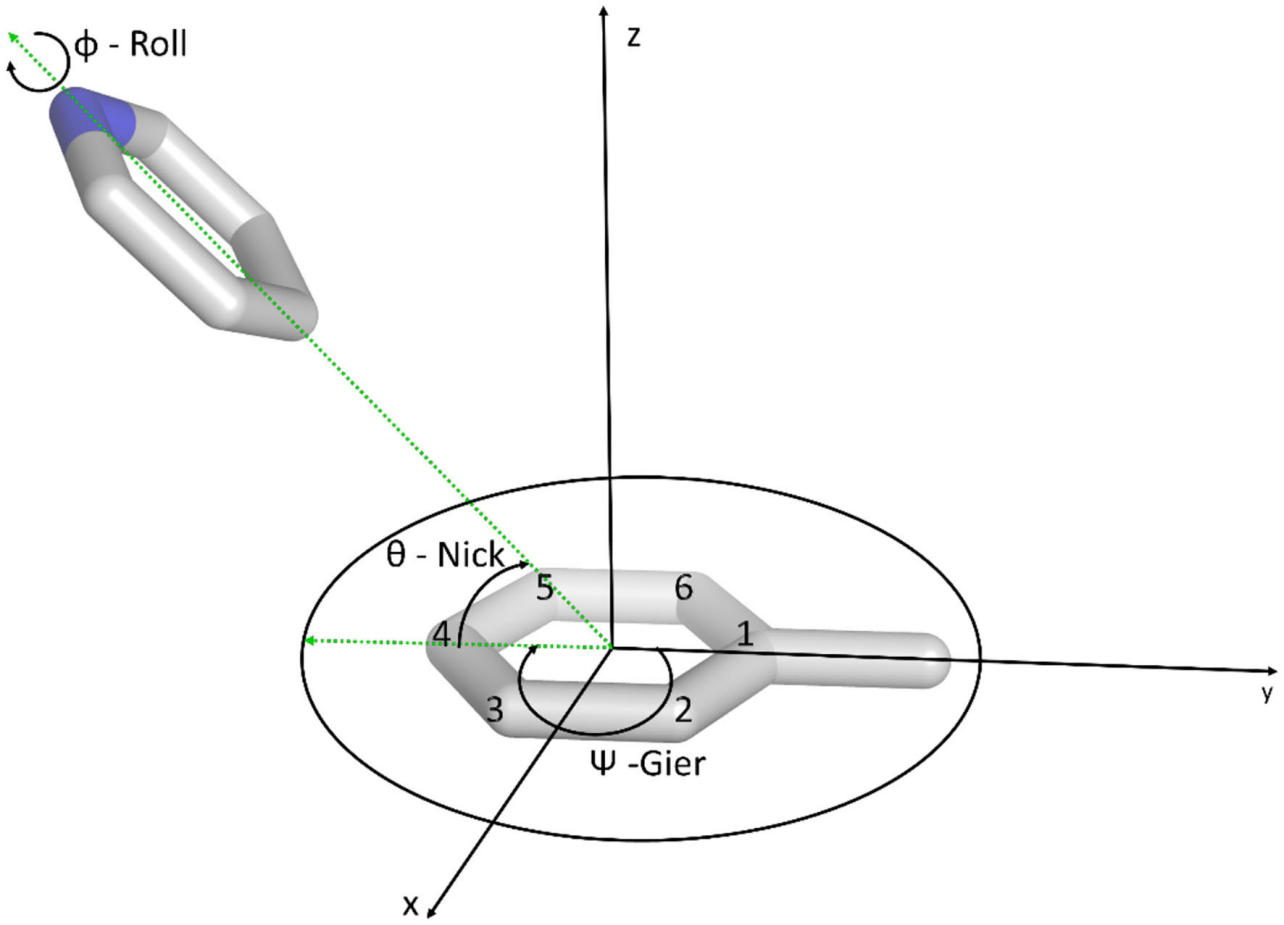
Definition of the coordinate system and the Tait-Bryan angles used in the analysis process. The origin of the coordinate system is defined as the center of the benzene ring of toluene.

The orientation of the stacked molecule during the simulation relative to the reference was described in terms of the Tait-Bryan angles (Markley and Crassidis, 2014). We especially focused on the *nick* and *gier* angles, as shown in Figure 2. Therefore, a reference coordinate system was defined using the toluene orientation. The y-axis is positioned in the direction from the ring C4 atom (para position) to the methyl carbon atom (cf. Figure 2). The x-axis was initially positioned in the direction from the center of mass of the C2 and C3 to the center of mass of the C4 and C5 atoms. From these two vectors we calculated the z-axis as the resulting cross product. The direction was chosen to obtain a right-handed coordinate system. To ensure an orthogonal coordinate system we recalculated the x-axis as the cross product of the y- and z-axis. The origin of the coordinate system was defined as the center of mass (COM) of the aromatic ring of the toluene molecule.

We aligned the obtained trajectories on the toluene molecule and then transformed the coordinates of the stacking heteroaromatic molecule into the previously introduced coordinate system. Furthermore, we assigned a “nose” vector **r**. The atoms chosen for each molecule can be found in Supplementary Figure 1. The vector **r** was normalized to length 1, and the *nick angle* θ and *gier* angle Ψ were calculated as follows.

(2) $$\begin{array}{r} nick\;\left(\theta \right)=arcos\left(r_{z}\right)\cdot \frac{180}{\pi }-90 \end{array}$$

(3) $$\begin{array}{r} gier\;\left(\text{Ψ}\right)=\arctan\left(\frac{r_{x}}{r_{y}}\right)\cdot \frac{180}{\pi } \end{array}$$

These angles were used to describe the molecular orientation in reference to the toluene molecule. In all frames where the center of mass was in the negative z-direction, the z-component of **r** was reversed, corresponding to mirroring the molecule by the xy-plane, i.e., the plane of the aromatic toluene (cf. Figure 2). Free energy profiles of the nick and gier angles obtained from kernel density estimation (KDE) with a kernel width of 0.1 radians.

## Results

### Geometry Optimizations

**Figure 3:**
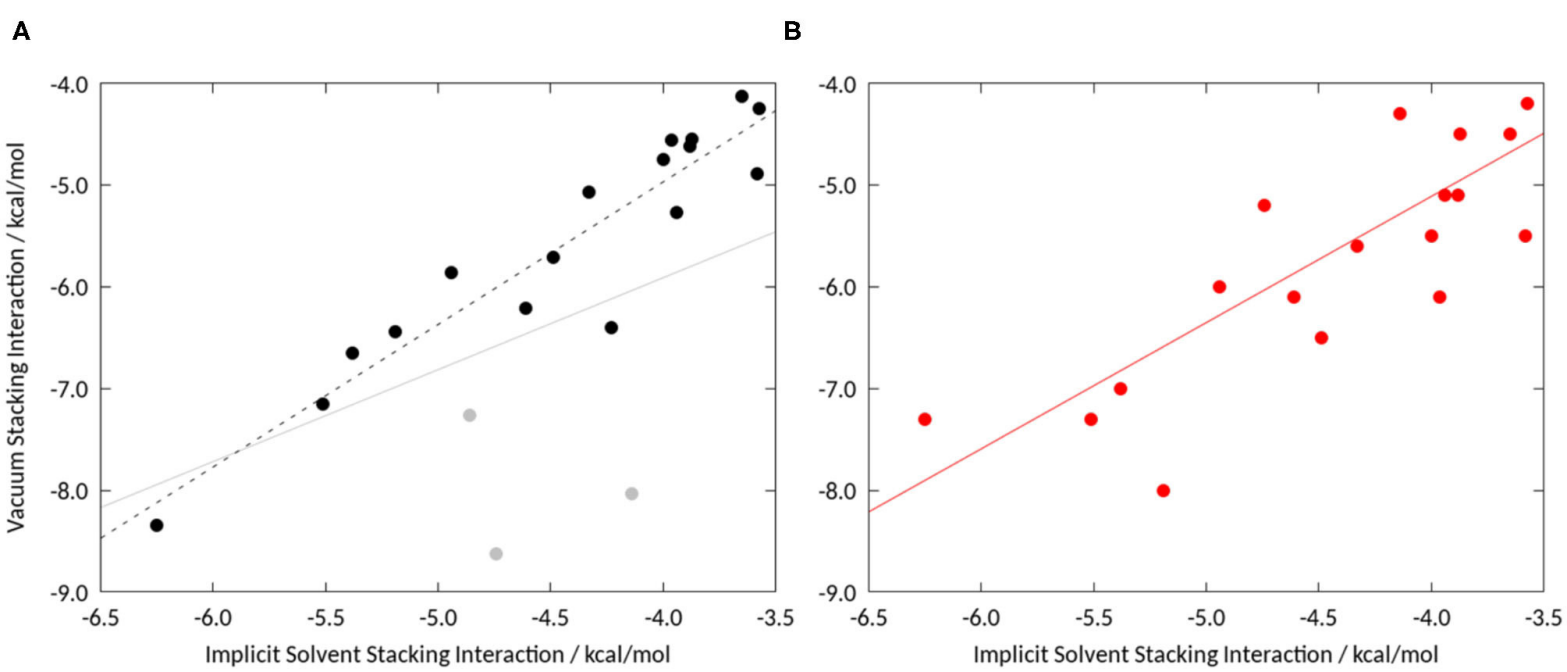
**(A)** Correlation of vacuum stacking interactions from an unrestrained geometry optimization with stacking interaction energies in implicit solvent: The Pearson correlation including the three compounds favoring T-stacked geometries (indicated in gray) is 0.74, without these three the correlation increases to 0.94. **(B)** Comparison of a grid-based approach by Bootsma et al. (2019) to identify minimum energy geometries with unrestrained geometry optimization in implicit solvent. We obtain a Pearson correlation of 0.77.

To assess the influence of solvation we initially performed unrestrained geometry optimizations, starting from the geometries provided by Bootsma et al. (2019), in implicit solvent using the quantum mechanical setup as described in the Methods section. We investigated the stacking interactions of a set of compounds that was recently studied in two publications on a truncated phenylalanine sidechain, i.e., toluene (Bootsma et al., 2019; Loeffler et al., 2020). Comparing the resulting stacking interaction energies, we find a Pearson correlation of 0.74 for the grid based approach (Bootsma et al., 2019) and 0.68 for the unrestrained energy optimizations (Loeffler et al., 2020). Comparing the obtained geometries, it is particularly striking that the compounds that prefer a T-stacked geometry in vacuum show a parallel displaced conformation in implicit solvent. If these compounds, (L09, L10, and L13), are excluded the correlation increases to 0.94 (see Figure 3A). This shows that even continuum models allow for different optimal stacking geometries compared, especially if T-stacked geometries are favored in vacuum.

Besides the high-level quantum mechanical calculations we performed simulations in water and vacuum using ANI (Smith et al., 2017) and the General Amber Force Field (GAFF) (Wang et al., 2004). To compare the resulting stacking interaction energies from ANI and GAFF with published QM data, we performed geometry optimizations of the complexes and of respective monomers (Supplementary Table 1). The stacking interaction was then calculated via the supermolecular approach, Equation (1) (Beljonne et al., 2000).

**Figure 4:**
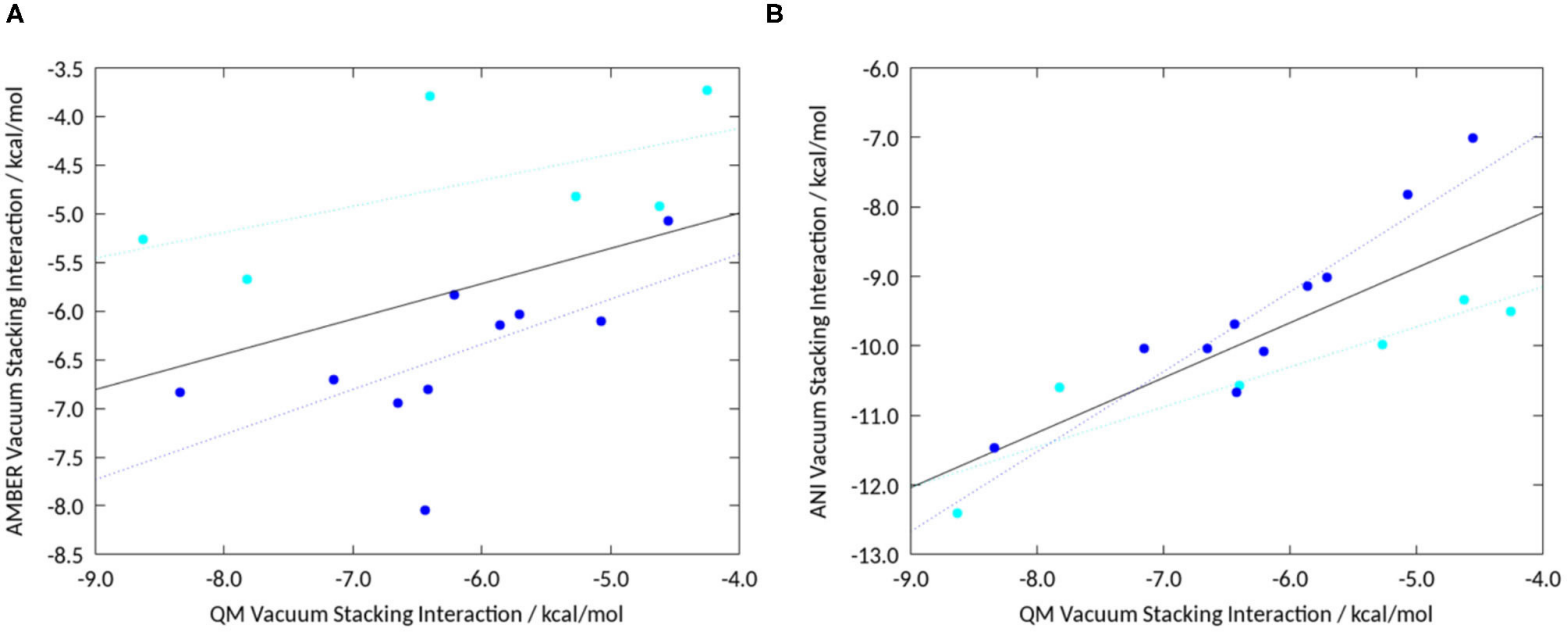
**(A)** Vacuum stacking interactions from geometry optimizations using GAFF correlated with high-level QM calculations. Overall the Pearson correlation is 0.41. **(B)** Correlation of stacking interaction energies calculated from geometry optimizations using ANI with published unrestrained geometry optimizations using high-level QM calculations (Loeffler et al., 2020). Shown in dark blue are the 6-membered rings, and in cyan the 5-membered heteroaromatics. We find an overall correlation of 0.81, the linear fit for all complexes is shown in black. The linear fit for the individual groups of compounds are shown in dark blue for the 6-membered rings and in cyan for the 5-membered rings, respectively.

For the GAFF stacking interactions, we obtained an overall Pearson correlation of 0.41, as shown in Figure 4A. The lack of correlation between GAFF and QM data emphasizes that stacking interaction of different heteroaromatics with benzene is not well-parametrized in classical force field-based approaches. Individually, for the 5-membered rings the correlation increases to 0.61 and for the 6-membered rings to 0.60, indicated by the cyan and dark blue line in Figure 4A. The overall Pearson correlation for our set of compounds of QM vacuum stacking interactions with ANI stacking interactions results in 0.81. By only taking the 6 membered rings into account, the correlation increases to 0.93, depicted by the dark blue line in Figure 4B. For the 5-membered rings alone the correlation results in 0.91 (Figure 4-cyan line). The comparison between the results obtained with different methods is summarized in Supplementary Table 1.

### Molecular Dynamics Simulations

As starting geometries for the molecular dynamics simulations we used the optimized structures published by Bootsma et al. (2019), and solvated these structures as described in the Methods section. The geometries by Bootsma et al. (2019), were obtained by performing elaborate high-level quantum mechanical calculations. It has been shown that the potential energy surface of stacked heteroaromatics is rather shallow, therefore, we focused our analysis on the relative orientation of the respective heteroaromatic rings rather than x,y, and z coordinates. Thus, we analyzed the trajectories using the relative orientations of the stacked heteroaromatics to toluene, i.e., the nick and the gier angle, as described in the Methods section. We highlight four systems in these sections, additional plots can be found in the Supplementary Material.

In general, we can see that the nick angle shows less variation than the gier angle regardless if the simulation is performed in vacuum or water (cf. Supplementary Figure 4). However, comparing the individual systems, either simulated in vacuum or water, different population distributions can be observed.

For the benzene-toluene complex, we sample both the π-π stacked and the T-stacked conformations (cf. Supplementary Figure 5). However, we can see a clear preference for the π-π stacked geometry in vacuum and explicit solvation. The T-stacked geometry can only be found stabilized in simulations using explicit solvent. However, even in the simulations performed in vacuum, we can show that the two molecules are hardly ever completely parallel, but almost always slightly tilted (Supplementary Figure 1), a fact that is very difficult to include in grid-based approaches using single point calculations.

**Figure 5:**
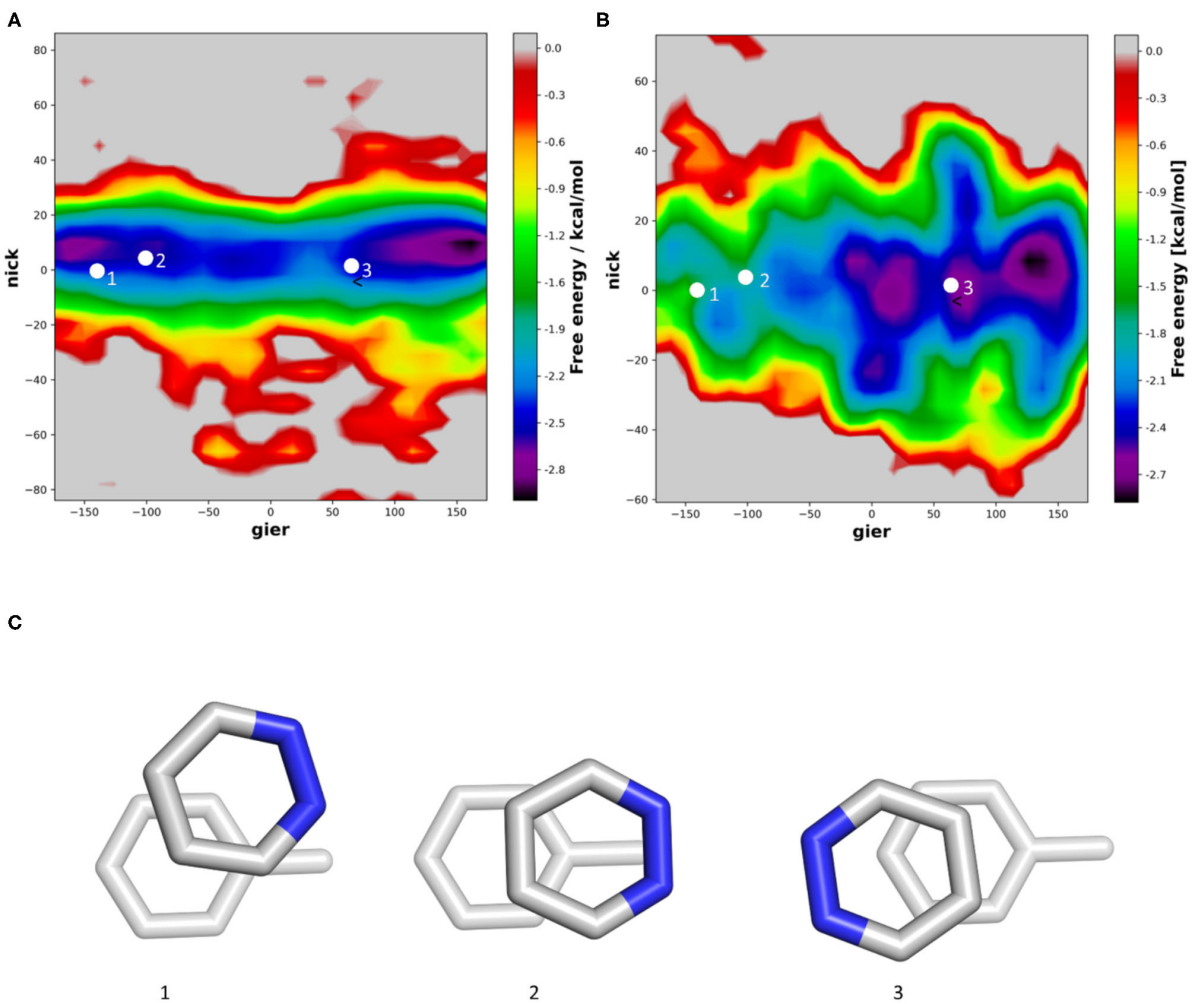
2D histogram analysis of the nick and gier angles of pyrazine in molecular dynamics simulations stacked with toluene. Simulations were performed in vacuum **(A)** and using explicit solvation **(B)**. We projected the orientations from published geometry optimizations in vacuum **(C)** into the density surface.

In contrast to benzene, pyridazine has a substantial dipole, due to the two neighboring heteroatoms. In vacuum, we can clearly observe that the orientations proposed from QM simulations represent the two main minima (Figure 5A). In our trajectories, the main orientation is found when the two dipoles are aligned but pointing into opposite directions (Figure 5C). In the presence of a solvent, no deep minimum can be identified, but we can clearly see, that an orientation in which the two Nitrogen atoms are orientated directly toward the methyl group of toluene is substantially less likely (Figure 5B). This is well in line with previously published results, where a second minimum was identified in implicit solvent geometry optimization (Loeffler et al., 2020). In the violin plots (Supplementary Figure 4), we can see that in the gier angle the distribution of the minima is ~30°, which corresponds to a rotation by one aromatic bond of the aromatic ring.

**Figure 6:**
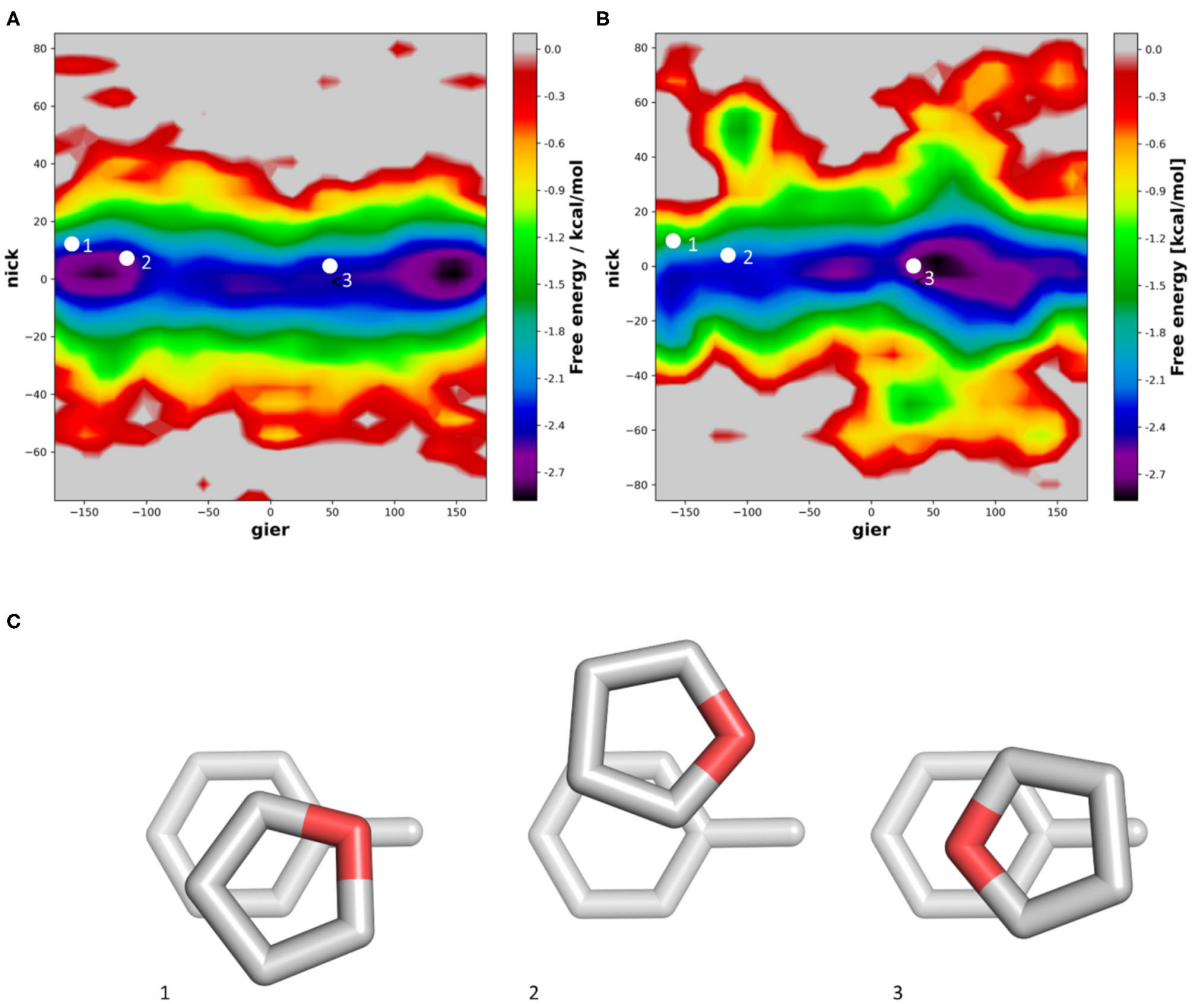
Distribution of the nick and gier angles of furan during molecular dynamics simulations in complex with toluene in vacuum **(A)** and in water **(B)** using 2D histograms. We mapped the orientations of published optimized geometries of furan stacking with toluene **(C)** into the density surface.

For five-membered rings, the inserted heteroatoms play a crucial role for the stacking interaction strength and conformations. In the example of furane we can find one orientation sampled very commonly. As mentioned previously, vacuum quantum mechanical calculations show low energy conformations when the dipole of furan and toluene are aligned. In our simulations we find that this orientation is indeed favorable, when performing the simulations in vacuum (Figure 6A). However, when performing the simulations in water, we can clearly observe a shift in the population (Figure 6B). In the violin plot (Supplementary Figure 4), this population shift is especially visible in the nick angle, clearly showing a more favorable tendency for T-stacked geometries in water compared to the vacuum distributions. Similar to the simulations of pyrazine, we can now identify the most favored orientation where the Oxygen atom is orientated toward the solvent rather than the methyl group of toluene (Figure 6C). This conformation is stabilized by the surrounding solvent. Furthermore, we can observe a slightly higher occurrence of T-stacked geometries in water, which are also stabilized by interactions of the heteroatom and the aromatic π-cloud with surrounding water molecules.

**Figure 7:**
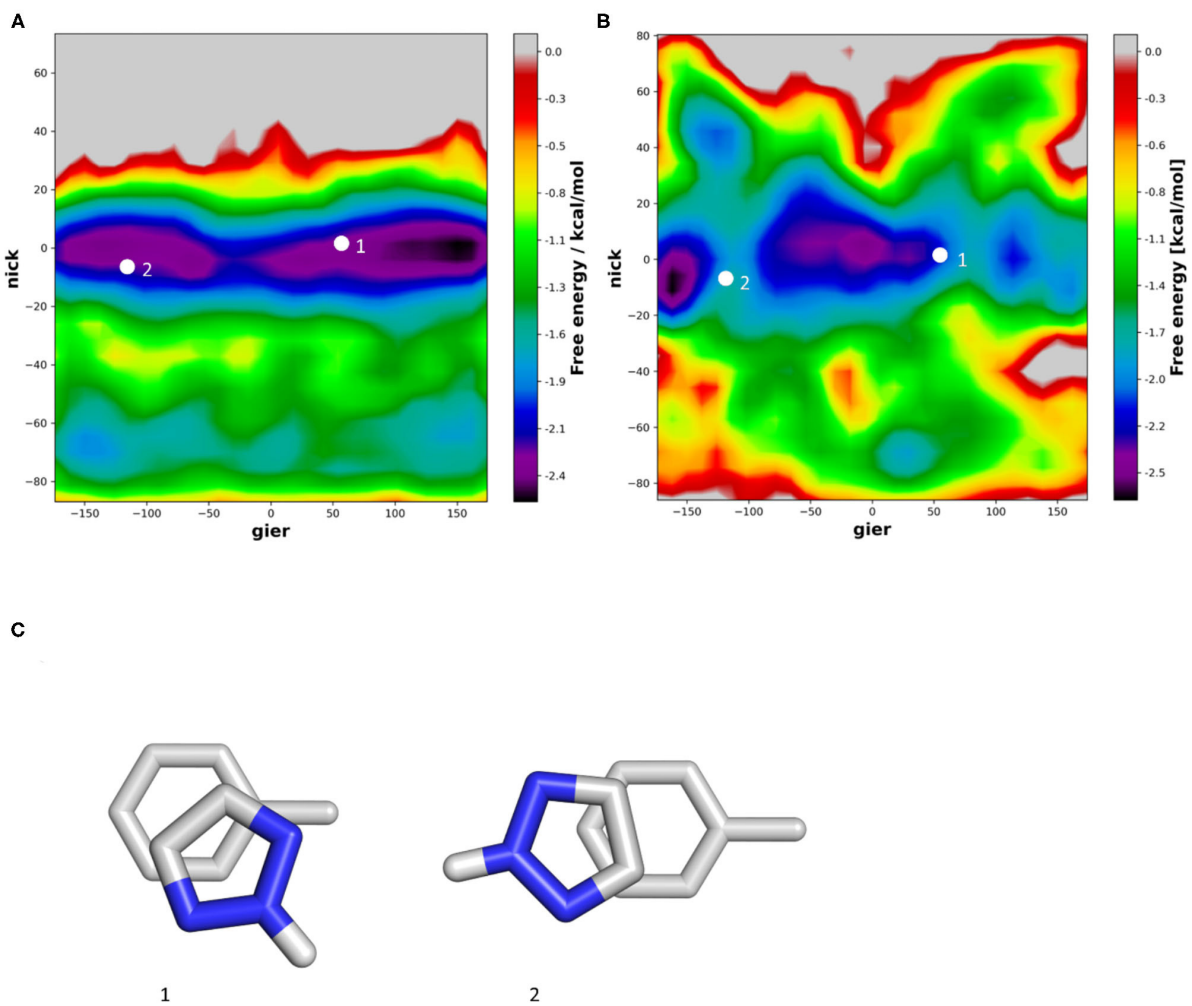
2D-histogram analysis of the nick and gier angles of triazole during the molecular dynamics simulations of the stacking interactions with toluene in vacuum **(A)** and in water **(B)**. **(C)** Shows the optimized geometries obtained from a grid-based optimization approach in vacuum.

Introducing a protonated Nitrogen atom to a five membered heteroaromatic system substantially influences its electrostatic properties and thereby stacking interaction (Bootsma et al., 2019). In our simulations we do not only see π-stacking but also various conformations of T-stacking. In vacuum, the T-stacking is observed exclusively as an interaction of the protonated Nitrogen atom with the toluene π-cloud (Figure 7A). During the simulations performed in water we additionally capture a conformation where the protonated Nitrogen atom interacts with the surrounding water molecules while the stacking interaction occurs between one of the carbon-bound hydrogen atoms (Figure 7B). Despite the different stacking geometries, we are able to identify a preference of orientation. In vacuum the strong dipole of triazole is aligned with the toluene dipole, while in water it is clearly favorable for the protonated Nitrogen atom to be orientated away from the methyl group of toluene, thereby allowing an improved interaction with the surrounding water molecules. These observations can also be confirmed in the violin plots (Supplementary Figure 4), where the distribution of the nick angles is substantially broader, indicating the occurrence of different T-stacked geometries.

## Discussion

In this study we performed molecular dynamics simulations of heteroaromatics, stacking with toluene in vacuum and in explicit solvent. It has been shown previously, that even implicit solvation can influence stacking interaction energies and geometries. In our results we observe this most prominently for heterocycles where a protonated Nitrogen atom is present. In vacuum, T-stacking is almost always favored in unrestrained geometry optimizations, while the parallel displayed geometry is more favorable when using an implicit solvent. Furthermore, we also calculated the vacuum stacking interactions by using ANI. Overall, we find a good correlation of the resulting energies with DFT calculations, despite an offset in the absolute energy values (see Figure 3). However, for the 5-membered rings, three complexes reveal a substantially stronger stacking interaction with ANI, namely furan, isoxazole, and oxazole. If these three complexes are neglected, the correlation increases to 0.93. This might indicate that the Oxygen atom in aromatic rings is not yet perfectly trained within the ANI network to characterize such subtle intermolecular interactions.

**Figure 8:**
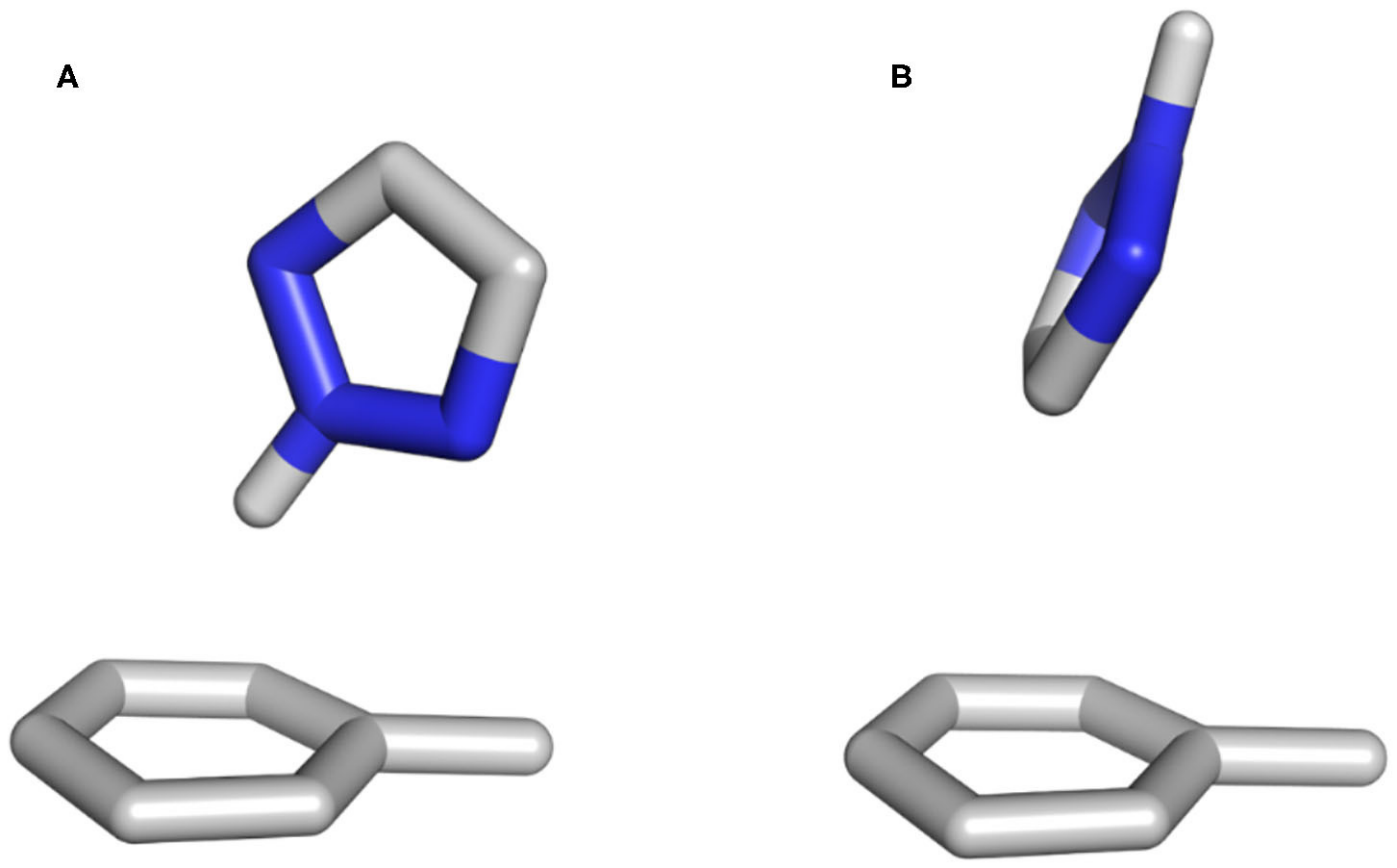
Two different T-stacked conformations identified in the simulations using explicit solvent. The geometry shown in **(A)** can also be found in the vacuum simulations. The conformation in **(B)** however, can only be sampled when using explicit solvation, as it needs to be stabilized by the surrounding water molecules.

Previous publications have shown that vacuum stacking interactions are stronger when heteroatoms are positioned outside the toluene π-cloud (Huber et al., 2014; Bootsma et al., 2019). When checking the position of the heteroatoms during our simulations, we can confirm for pyrazine that in both vacuum and water the Nitrogen atoms are outside the underlying toluene for more than 70% of the frames. However, as the system reveals a high flexibility, the nitrogen atoms can also be found oriented toward the π-cloud. The vacuum simulations of furan show that the oxygen atom is favorable outside the π-cloud in ~70% of the simulation. This even increases to more than 80% for the simulation in water, where the oxygen atom of furan can interact with the surrounding water molecules. In the case of triazole, this observation could not be confirmed in vacuum. On the one hand, the protonated Nitrogen atom of triazole is the main interaction partner for the T-stacked geometries (Figure 8A), and on the other hand, in vacuum, the positive polarization of the protonated Nitrogen atom is the only possible interaction partner for the π-cloud of the underlying toluene. The influence of solvation was not only visible from our molecular dynamics simulations, but also from the geometry optimizations using implicit solvation. In contrast to the optimization performed in vacuum, the unrestrained optimization using implicit solvation resulted in a π-π stacked geometry rather than a T-stacked geometry. However, the protonated Nitrogen atom group is still positioned inside the π-cloud. Our simulations in water show that for more than 65% of all frames the protonated Nitrogen atom group is located outside of the π-cloud, interacting with the surrounding water molecules. Additionally, we can identify two different T-stacked conformations in our simulations in water as shown in Figures 7B, 8. On the one hand, we observe a T-stacked geometry stabilized by the interaction of the protonated Nitrogen atom with the underlying π-cloud (Figure 8A). This geometry can be seen in vacuum as well as in explicit solvent simulations (Figure 7). On the other hand, we identify a T-stacked geometry where the protonated Nitrogen does not interact with the π-cloud but rather with the surrounding water molecules (Figure 8B).

ANI allows to explore the conformational space of organic molecules at lower computational cost and facilitates the characterization and understanding of non-covalent interactions i.e., stacking interactions and hydrogen bonds. Nevertheless, in its current form ANI cannot be used to analyze protein-ligand interactions, as the ANI potentials are not yet parametrized for proteins. Furthermore, the water molecules in ANI still need to be evaluated and compared to classical water models, e.g., OPC, SPC, and the TIP water models. Future work on ANI will aim to develop and include new methods to better describe long-range interactions by including coulomb interactions. The constant addition of more data to machine learning methods will make ANI even more generalizable and improve calculations in different chemical environments, the treatment of ions and the applicability to describe reactions (Smith et al., 2019).

In this study we can show that by using neural networks we can get information not only on geometries but also on intermolecular interactions correlating well with state-of-the-art QM calculations. Furthermore, using neural networks we now can generate ensembles of stacked heteroaromatic complexes including explicit solvation. Both of these points can give crucial information in the early stages of computational drug design.

## Conclusion

In our study we investigated the influence of solvation on complexes of stacked heteroaromatics using implicit solvent geometry optimizations and molecular dynamics simulations including explicit solvation. We demonstrate that potentials derived from machine learning can be used to perform molecular dynamics simulations as the geometries obtained using high level quantum mechanical simulations are present within the ensemble in solution with shifted populations. Additionally, the calculated stacking interactions using neural networks energies calculated in vacuum correlate well with high level quantum mechanical calculations. However, heterocycles containing an oxygen, i.e., furan, oxazole and isoxazole are overpredicted in terms of stacking interaction energies. The ensembles from the molecular dynamics simulations are well in line with previously published results and show that heteroatoms are in general favorable outside of the π-cloud. This is true for heteroatoms except for secondary amines, which, especially in vacuum, show beneficial interactions with the underlying π-cloud. Furthermore, we highlight the necessity of including solvation properties of aromatic molecules as the optimal geometries can differ substantially depending on whether water molecules are present as possible interaction sites or not. The effect of population shifts naturally increases with the polarity of the aromatic ring and is especially notable if secondary amines are present.

## Data Availability Statement

The original contributions presented in the study are included in the article/Supplementary Material, further inquiries can be directed to the corresponding author.

## Author Contributions

The manuscript was written through contributions of all authors. All authors have given approval to the final version of the manuscript.

## Conflict of Interest

The authors declare that the research was conducted in the absence of any commercial or financial relationships that could be construed as a potential conflict of interest.

## Abbreviations

ANI: Accurate NeurAl networK engINe for Molecular Energies
GAFF: General Amber Force Field
MD: Molecular Dynamics
QM: Quantum Mechanics
SAR: Structure Activity Relationship.
