# Supplementary material for: Conformational Shifts of Stacked Heteroaromatics: Vacuum vs. Water Studied by Machine Learning
Source: Front Chem. 2021 Mar 26;9:641610. doi: 10.3389/fchem.2021.641610 (PMC8032969; doi:10.3389/fchem.2021.641610)
Supplement: Supplementary file 1 [file Table_1.DOCX]

Supporting Information: Vacuum vs. Water: Population Shifts of Solvated Stacked Heteroaromatics using Machine Learning

Johannes R. Loeffler^†^, Monica. L. Fernández-Quintero^†^, Franz Waibl^†^, Patrick Quoika^†^, Florian Hofer^†^, Michael Schauperl^†^, Klaus R. Liedl^†^*

Corresponding Author: * [klaus.liedl@uibk.ac.at](mailto:klaus.liedl@uibk.ac.at)

Table S1: Comparison of vacuum stacking interaction energies of unrestrained high level quantum mechanical, GAFF and ANI-1 geometry optimizations.

| Heterocycle | Stacking QM / kcal/mol | Stacking FF / kcal/mol | Stacking ANI-1 / kcal/mol |
| --- | --- | --- | --- |
| Furan | -4.25 | -3.73 | -9.50 |
| Isooxazole | -5.27 | -3.70 | -9.98 |
| Oxazole | -4.62 | -3.56 | -9.34 |
| Pyrazole | -6.40 | -4.82 | -10.57 |
| Triazole | -7.82 | -4.03 | -10.60 |
| Tetrazole | -8.63 | -4.08 | -12.41 |
| Benzene | -4.55 | -4.92 | -7.01 |
| Pyridine | -5.07 | -4.81 | -7.83 |
| Pyrazine | -5.86 | -4.65 | -9.14 |
| Pyrimidine | -6.21 | -3.79 | -10.08 |
| Pyridazine | -5.71 | -3.79 | -9.01 |
| 1,2,5-Triazin | -6.65 | -3.79 | -10.03 |
| Triazin | -7.15 | -5.68 | -10.04 |
| 1,2,4,5-Tetrazin | -6.42 | -5.37 | -10.67 |
| Tetrazin | -6.44 | -5.68 | -9.69 |
| Pyrimidone | -8.34 | -5.26 | -11.47 |


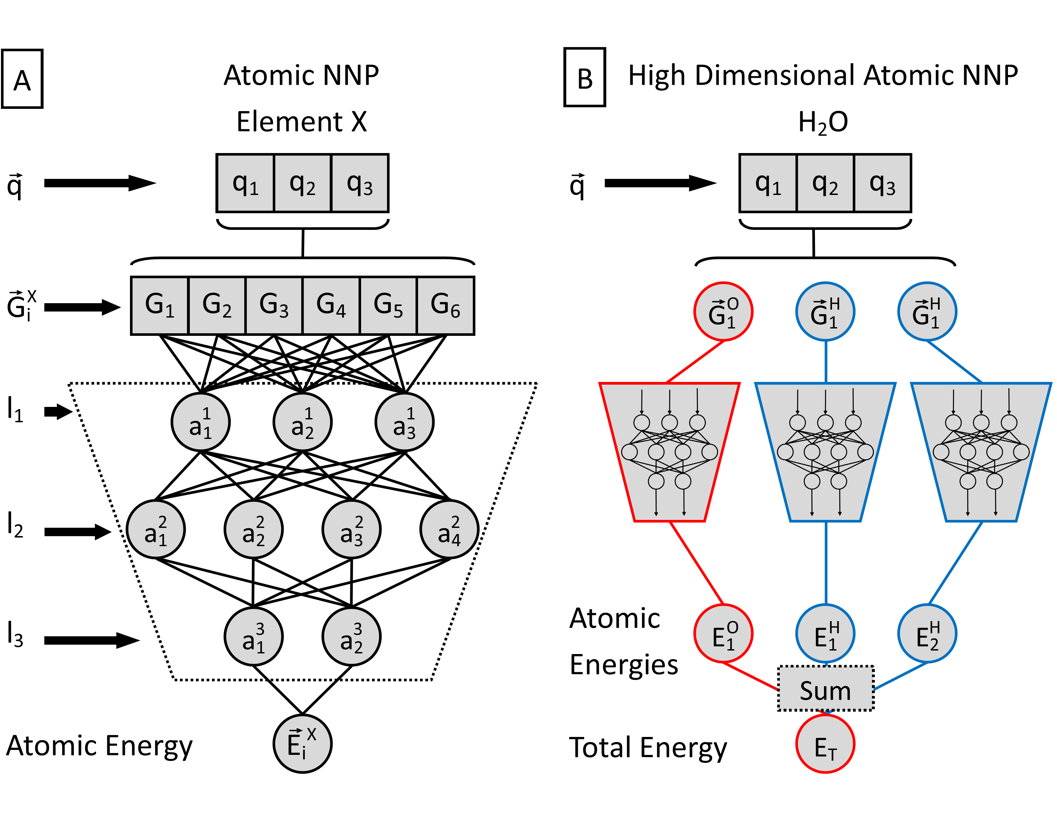


*Figure S1: Schematic representation of Behler and Parinellos atomic NNP or HD-atomic NNP. A: The*

*schematic algorithm of an atom-specific neural network potential. The input coordinates (*$\vec{q}$*) are transformed*

*to yield the atomic eigenvector,*$\vec{G_{i}^{X}}$*. This eigenvector is fed into the neural network to predict the total*

*energy* $E_{i}^{X}$ *B: The high-dimensional atomic NNP for a water molecule. The atomic eigenvector is calculated*

*for each atom individually to produce the respective energy* $E_{i}^{X}$ *. The total energy*$E_{\tau}$ *is calculated as the*

*sum of the individual energies* $E_{i}^{X}$ .


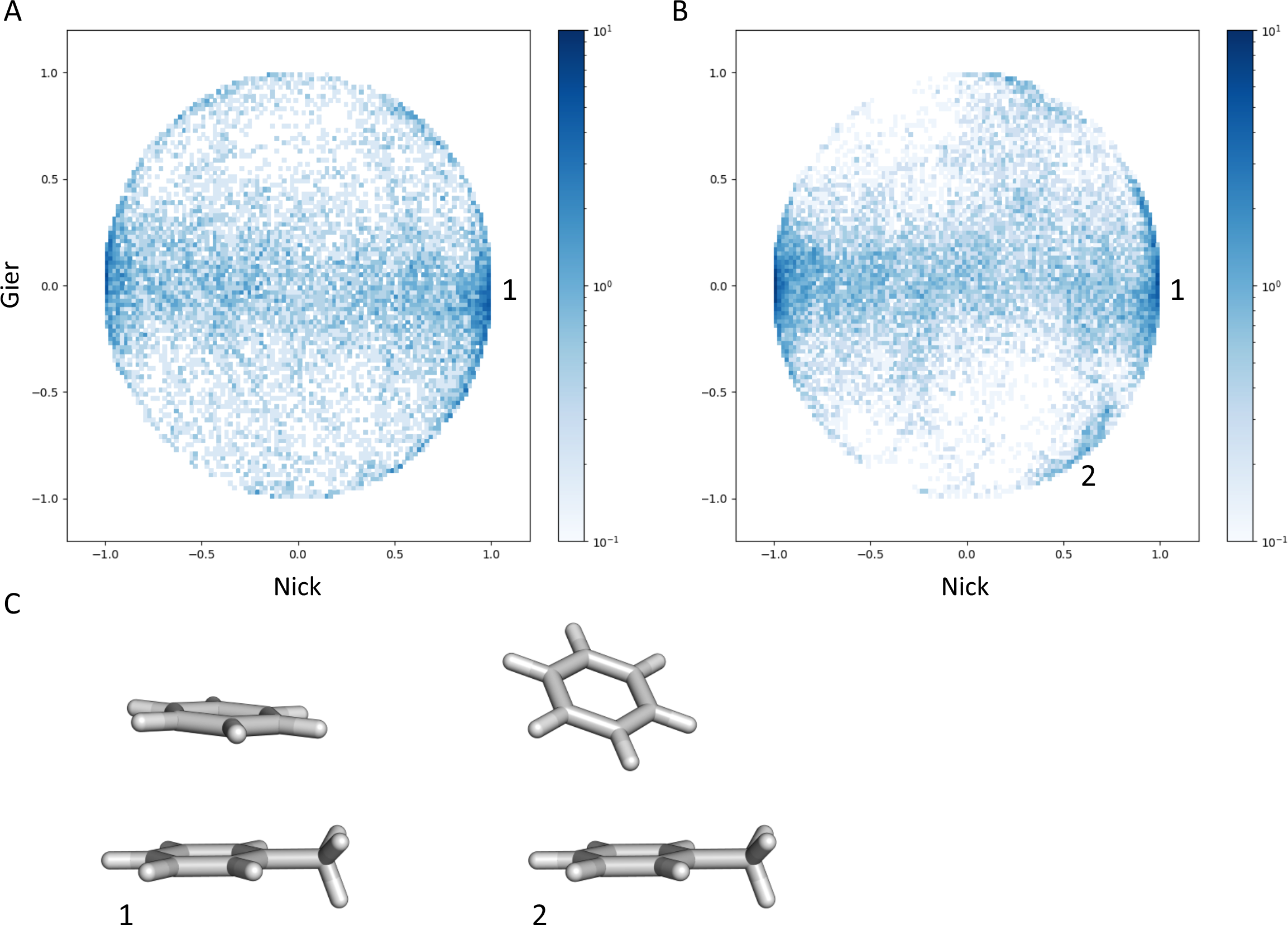


Figure S2: Nick and gier angle distributions in vacuum (A). T-stacking is sampled but not stabilized. Simulation in water (B) reveals a stabilized T-stacked geometry. Representative structures for π-π stacking and the stabilized T-stacked geometry (C).


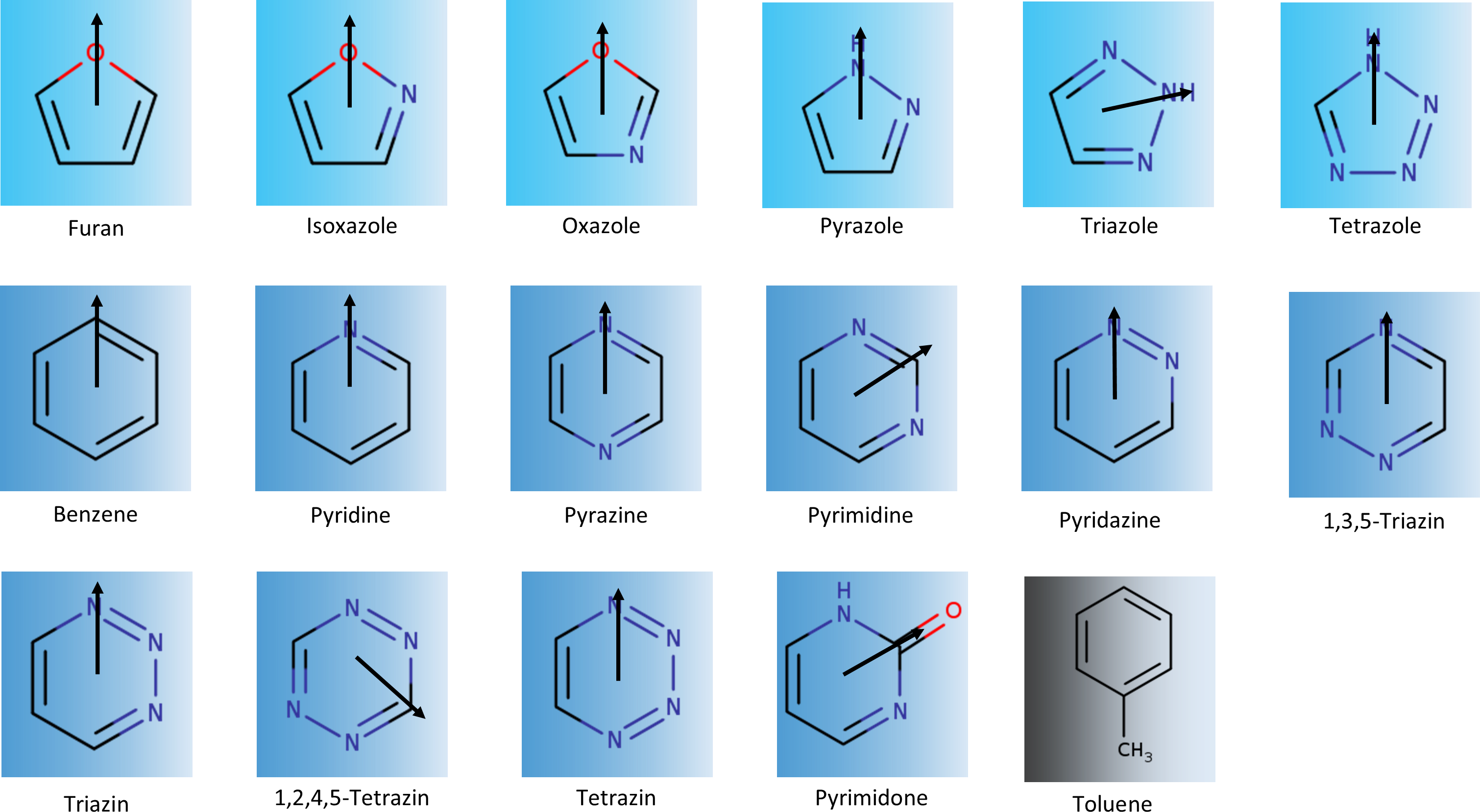


Figure S3: Definition of the nose vector for all investigated heteroaromatics


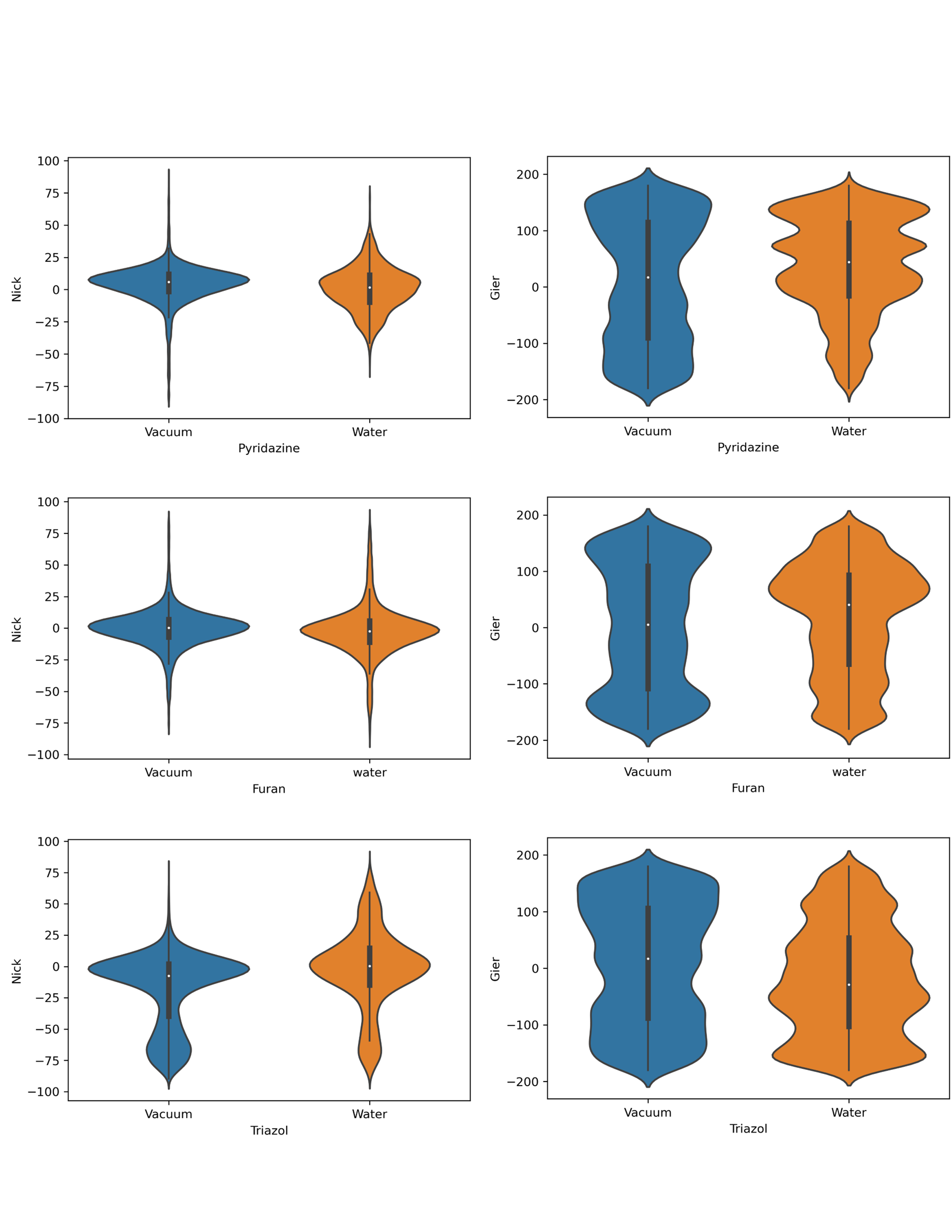


Figure S4: Violin Plots showing the distribution of nick and gier angles during the simulations for Pyridazine, Furan, and Triazol.

.
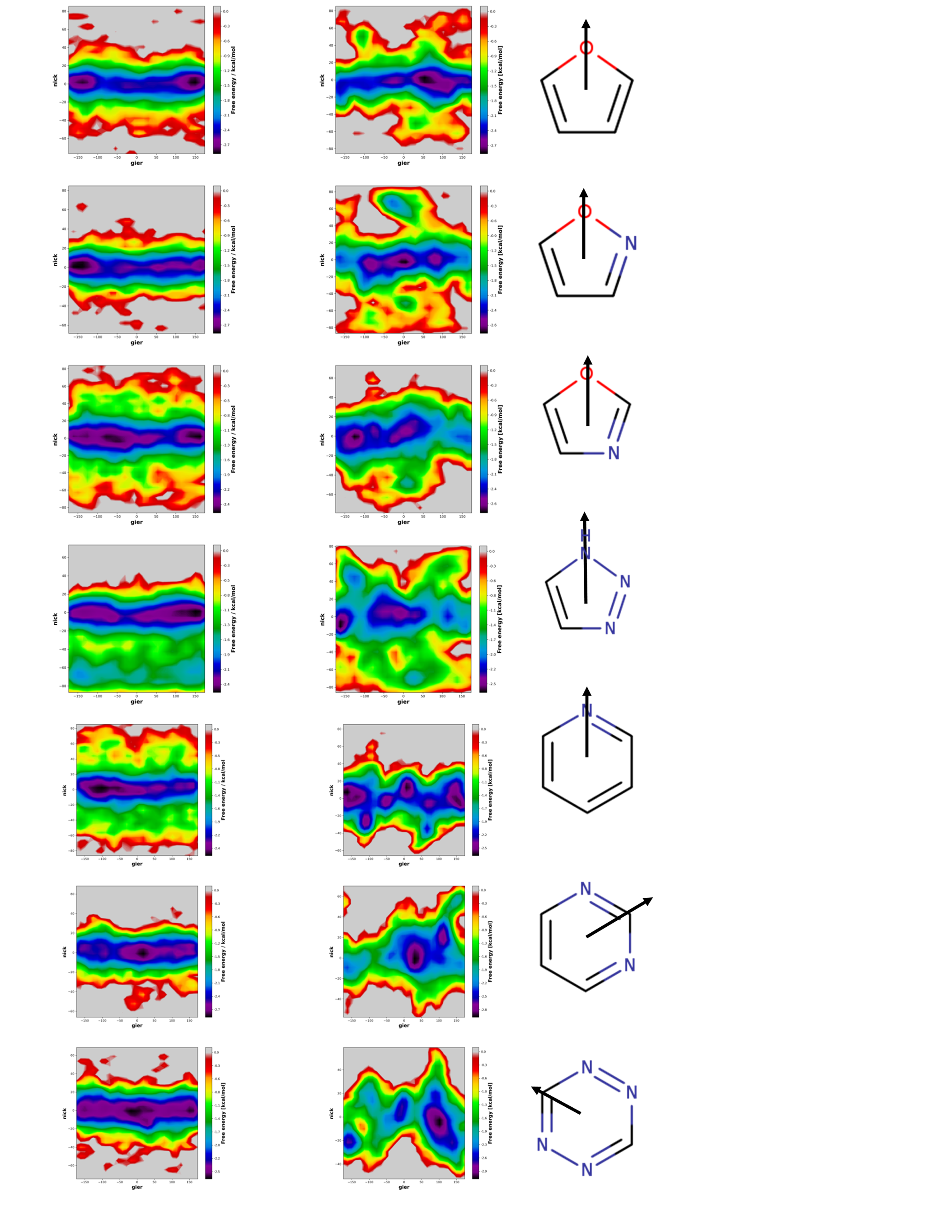


Figure S5: Free energy surfaces depending on the nick and gier angles for additional systems. In the first column, simulations in vacuum, in the second column simulations in water and in the third columns the respective heteroaromatic compound including the nose vector.
